# Modulation of Gut Microbiota by Low Methoxyl Pectin Attenuates Type 1 Diabetes in Non-obese Diabetic Mice

**DOI:** 10.3389/fimmu.2019.01733

**Published:** 2019-07-30

**Authors:** Chengfei Wu, Li-Long Pan, Wenying Niu, Xin Fang, Wenjie Liang, Jiahong Li, Hongli Li, Xiaohua Pan, Wei Chen, Hao Zhang, Jonathan R. T. Lakey, Birgitta Agerberth, Paul de Vos, Jia Sun

**Affiliations:** ^1^State Key Laboratory of Food Science and Technology, Jiangnan University, Wuxi, China; ^2^School of Food Science and Technology, Jiangnan University, Wuxi, China; ^3^School of Medicine, Jiangnan University, Wuxi, China; ^4^Department of Surgery, University of California, Irvine, Orange, CA, United States; ^5^Division of Clinical Microbiology, Department of Laboratory Medicine, Karolinska Institute, Karolinska University Hospital, Huddinge, Sweden; ^6^Division of Medical Biology, Department of Pathology and Medical Biology, University of Groningen, University Medical Center Groningen, Groningen, Netherlands

**Keywords:** low-methoxyl pectin, autoimmune diabetes, immunomodulation, gut microbiota, intestinal homeostasis

## Abstract

Intestinal homeostasis underpins the development of type 1 diabetes (T1D), and dietary manipulations to enhance intestinal homeostasis have been proposed to prevent T1D. The current study aimed to investigate the efficacy of supplementing a novel specific low-methoxyl pectin (LMP) dietary fiber in preventing T1D development. Female NOD mice were weaned onto control or 5% (wt/wt) LMP supplemented diets for up to 40 weeks of age, overt diabetes incidence and blood glucose were monitored. Then broad-spectrum antibiotics (ABX) treatment per os for 7 days followed by gut microbiota transfer was performed to demonstrate gut microbiota-dependent effects. Next-generation sequencing was used for analyzing the composition of microbiota in caecum. Concentration of short chain fatty acids were determined by GC-MS. The barrier reinforcing tight junction proteins zonula occludens-2 (ZO-2), claudin-1 and NOD like receptor protein 3 (NLRP3) inflammasome activation were determined by Western blot. The proportion of CD25^+^Foxp3^+^CD4^+^ regulatory T cell (Foxp3^+^ Treg) in the pancreas, pancreatic and mesenteric lymph nodes was analyzed by flow cytometry. We found that LMP supplementation ameliorated T1D development in non-obese diabetic (NOD) mice, as evidenced by decreasing diabetes incidence and fasting glucose levels in LMP fed NOD mice. Further microbiota analysis revealed that LMP supplementation prevented T1D-associated caecal dysbiosis and selectively enriched caecal bacterial species to produce more SCFAs. The LMP-mediated microbial balance further enhanced caecal barrier function and shaped gut-pancreatic immune environment, as characterized by higher expression of tight junction proteins claudin-1, ZO-2 in caecum, increased Foxp3^+^ Treg population and decreased NLRP3 inflammasome activation in both caecum and pancreas. The microbiota-dependent beneficial effect of LMP on T1D was further proven by the fact that aberration of caecal microbiota by ABX treatment worsened T1D autoimmunity and could be restored with transfer of feces of LMP-fed NOD mice. These data demonstrate that this novel LMP limits T1D development by inducing caecal homeostasis to shape pancreatic immune environment. This finding opens a realistic option for gut microbiota manipulation and prevention of T1D in humans.

## Introduction

Type 1 diabetes (T1D) is characterized by autoreactive T cell-mediated selective destruction of pancreatic beta-cells in genetically predisposed individuals (1). Despite its genetic attribution, accumulating evidence supports that intestinal homeostasis (2, 3), which involves the complex interaction between host immunity and commensal microbiota, is crucial for shaping the pancreatic immune environment and modulating the development of T1D (3–5).

Gut microbiota affects not only fermentation of food components and production of metabolites (5) but also influences host immune status and gut permeability, thus impacting autoimmune processes during T1D (6). It has been proven that when the mutualistic relation between host immunity and the microbiota is compromised, alterations in bacteria function and diversity, a process called dysbiosis, may cause or contribute to T1D development (2, 3, 6, 7). For example, an increased abundance of *Bacteroidetes* at the phylum level and other types of gut dysbiosis has been regarded as a hallmark of T1D onset (8, 9). Gut dysbiosis is frequently associated with gut barrier dysfunction, resulting in increased gut permeability and translocation of diabetogenic proteins and macromolecular structures in T1D susceptible animal models and in humans to contribute to the disease development (4, 10, 11). The same studies showing the involvement of gut dysbiosis in T1D also demonstrate the flexibility of the gut microbial communities for manipulation by food components, which opens new venues to treat or prevent T1D (5, 9, 12).

One way to influence the microbiota is by providing them with dietary carbohydrate fibers that support microbiome diversity and growth of beneficial gut bacteria (12, 13), and to steer the aforementioned intestinal dysfunction (14–16). We have earlier shown that only dietary fibers with specific chemical compositions have these beneficial effects, while others are ineffective (16, 17). Finding dietary fibers with efficacy in preventing intestinal barrier dysfunction and dysbiosis is therefore highly needed as they might be effective in suppressing T1D development.

Originated from fruit and vegetables, pectin is a family of carbohydrates with a complex structure with an α (1–4)-linked galacturonic acid polysaccharide backbone and can differ in degree of methyl esterification. Low-methoxyl pectin (LMP) refers to pectin in which the acid units in the backbone are methyl-esterified for <50%. Pectin has recently been shown to exhibit effects on intestinal microbiota and barrier function (14, 15). In addition, pectin or its fermentation products, such as acidic oligosaccharides in early life can result in the prevention of a number of allergic conditions and atopic dermatitis (18, 19). We have earlier shown that in the intestine, pectin, especially LMP, exert direct anti-inflammatory effects and reduce intestinal permeability (14, 15), and could therefore be instrumental in protecting against T1D.

In the present study, we investigated whether LMP supplementation influenced T1D development in non-obese diabetic (NOD) mice. Effects of LMP on diabetic incidence, aberration of pancreatic immune environment including NLPR3 inflammasome activation, and associated immune cell infiltration in NOD mice were studied. Also, effects on preventing T1D associated barrier dysfunction and dysbiosis were assessed (20). To determine the cause or consequence of LMP induced microbiota changes, microbiota was depleted by broad-spectrum antibiotics (ABX) treatment with or without LMP treated microbiota transferred to NOD mice with newly onset T1D, after which T1D development was followed.

## Materials and Methods

### High Performance Gel Permeation Chromatography (HPGPC)

The molecular weight of LMP was analyzed by HPGPC with an ultrahydrogel TM Linear column (300 mm × 7.8 mmid × 2). A Waters 1525 system with a RI detector was used to perform the experiment. The mobile phase was 0.1 N NaNO_3_ at a flow rate of 0.9 mL/min.

### High Performance Anion Exchange Chromatography With Pulsed Amperometric Detection (HPAEC-PAD)

LMP hydrolysis was carried out by adding 20 drops of 2 N trifluoroacetic acid into 1% (w/v) solution in DI water and heated at 95°C with shaking at 100 rpm in a shaking water bath for 6 h. The hydrolysates were filtered through 0.2 um filter and injected to an ICS-5000 system (Dionex, Sunnyvale, CA, USA) equipped with a Dionex CarboPac-PA20 analytical column (3 × 150 mm) (HPAEC-PAD) using gradient elution at a 0.5 mL/min flow rate.

### Fourier Transform Infrared Spectroscopy (FTIR)

LMP powder was desiccated in a desiccator before analysis by placing on an attenuated total reflectance sampling accessory (Smart iTR, Thermo Fisher Scientific, Waltham, MA, USA) of an FTIR spectrometer (Nicolet 6700, Thermo Fisher Scientific) equipped with a single bounce diamond crystal. FTIR spectra of the sample were obtained by co-adding 64 scans at the resolution of 4 cm^−1^ in mid-infrared region (4,000–400 cm^−1^).

### Animals, Diets, and Blood Glucose Measurements

Female NOD/LtJ mice were purchased from Su Pu Si Biotechnology Co., Ltd (Suzhou, Jiangsu, China) and maintained in SPF environment at the Animal Housing Unit of Jiangnan University under a controlled temperature and a 12-h light/12-h dark cycle. All animal experiments were carried out according to protocols approved by the Institutional Animal Ethics Committee of Jiangnan University (JN. No 20150331-0410). Four-weeks old female NOD mice were fed with AIN-93G purified diets or supplemented with 5% (wt/wt) LMP for up to 40 weeks of age (*n* = 20). The dosage was chosen based on previous studies (14, 21, 22), and this dose is within the reported range of LMP for nutritional supplementation (3.3–15%) in literature (23–25). Fasting blood glucose levels were monitored at 40th and 11th week and determined with an Accu-chek glycosometer (Roche Diagnostics, Almere, The Netherlands). A blood glucose level >14.0 mmol/L on two consecutive readings was considered to indicate a diabetic state.

### Histology

Pancreatic tissues were fixed, sectioned (5 μm thick) and stained with hematoxylin and eosin (H&E). The degree of insulitis was determined from multiple non-sequential slides from six individual mice. Slides were scanned by Pannormaic at 25× magnification. Mouse islets (30 per pancreata) were scored for the evaluation of insulitis and assigned a score by evaluating the degree of immune cell infiltration and categorized as follows: 0—no insulitis, 1—peri-insulitis, 2—insulitis with <50% infiltration of islets, 3—invasive insulitis with >50% in filtration of islets.

### ABX Treatment and Microbiota Transfer (MT)

For ABX treatment, mice were given 1 g/L metronidazole, 0.5 g/L vancomycin, 1 g/L ampicillin and 1 g/L neomycin (Sigma-Aldrich, St. Louis, MO, USA) by daily oral gavage of 200 μL of the ABX solution (100% H_2_O) for 7 days. In parallel with ABX treatment, mice were treated with 200 g/L amphotericin B of 100 μL in solution (100% H_2_O) to avoid occasional overgrowth of *Candida* spp. Following 7-days ABX treatment, to suppress spontaneous gut microbial growth, the two groups of mice, NOD+ABX and NOD+LMP+ABX received ABX treatment every week until the mice were sacrificed.

For MT experiments, caecal content from eight LMP-fed or normal chow-fed SPF donor NOD mice was collected and immediately mix sufficiently in anaerobic chamber (diluted 1:10 in a 50% glycerol/PBS solution). Next, the solution was divided into aliquots and frozen in liquid nitrogen and thereafter stored at −80°C. At the day of inoculation, caecal microbiota-suspensions were further diluted 1:3 prior to oral gavage (~1–5 × 10^8^ cells per mice, as determined by THOMA counting-chamber). Dependent on the different treatments described above, groups of mice were designated as NOD, NOD + ABX, NOD + ABX + MT, NOD + LMP, NOD + LMP + ABX, and NOD + LMP + ABX + MT.

### Western Blot

Tissue sample preparation and Western blotting were performed as previously described (16). Primary antibodies against NLRP3 (Cat: 15101), ASC (Cat: 67824), cleaved IL-1β (Cat: 63124), GAPDH (Cat: 5174), cleaved IL-18 (Cat: 52718) (all from Cell Signaling Technology, Beverly, MA, USA), caspase-1-p20 (Cat: sc-1597), ZO-2 (Cat: sc-11448), claudin-1 (Cat: sc-17658), and occludin (Cat: sc-8144) (Santa Cruz Biotechnology, Santa Cruz, CA, USA) were applied.

### Analysis of Short Chain Fatty Acids (SCFAs) and DNA Extraction for the Next-Generation Sequencing Analysis in Caecum

Caecal content samples (50 mg) were collected under sterile conditions and were stored in liquid nitrogen. Concentrations of SCFA were measured by GC coupled to the MS detector of GCMS-QP2010 (Shimadzu, Japan) as described previously (26). Bacterial genomic DNA from caecal content was extracted using FastDNA Spin kit for soil (MP Biomedicals, Santa Ana, California, USA) following the manufacturer's instructions. The V4 region of 16S rRNA was PCR-amplified using primers (sense: 5′-AYTGGGYDTAAAGNG-3′; antisense: 5′-TACNVGGGTATCTAATCC-3′). The PCR products were excised from a 1.5% agarose gel, purified by Gene Clean Turbo (MP Biomedicals, Santa Ana, CA, USA) and quantified by Quant-iT PicoGreen dsDNA Assay Kit (Life Technologies, Carlsbad, USA), following the manufacturer's instructions. Libraries were prepared using TruSeq DNA LT Sample Preparation Kit (Illumina, San Diego, USA) and sequenced for 500+7 cycles on Illumina MiSeq using the MiSeq Reagent Kit (500 cycles-PE).

### Isolation of Immune Cells From Pancreas, Pancreatic Lymph Node (PLN), and Mesenteric Lymph Node (MLN) of NOD Mice

Immune cells were freshly prepared from pancreas, pancreatic lymph node (PLN) and mesenteric lymph node (MLN) as previously described (27) by passing the freshly isolated organs through a 70 μm nylon mesh. Cells were then washed with cold PBS and resuspended in PBS for staining.

### Flow Cytometry

The single cells isolated from pancreas and lymph nodes were washed and resuspended in PBS. Surface staining was performed with the following mAbs: anti-CD45, -CD4, and -CD25 at room temperature for 30 min. Detailed antibody information was shown in Supplementary Table 1. Cells were then fixed and permeabilized. Intracellular detection of Foxp3 was performed using anti-Foxp3 at 4°C for 50 min.

### Statistical Analyses

Diabetes incidence was plotted according to the Kaplan-Meier method. Statistical significance was determined using a two-tailed Student's *t*-test or when comparing multiple groups, one-way analysis of variance followed by Tukey's *post-hoc* test. A *p*-value of <0.05 was considered statistical significant. Statistical analyses were carried out using GraphPad Prism 7 software (GraphPad, La Jolla, CA, USA).

## Results

### Chemical Characterization of LMP

**Figure 1:**
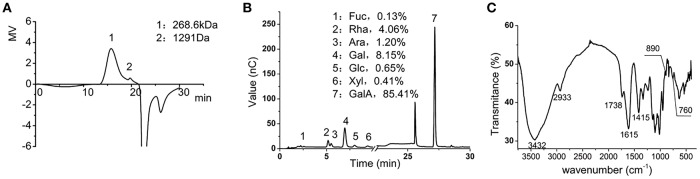
Chemical characterization of LMP. **(A)** The molecular weight of LMP was determined by HPGPC. **(B)** Chromatographic profile was obtained by HPAEC-PAD of monosaccharides of LMP. **(C)** FTIR profile of LMP.

We first determined the chemical composition of the LMP. The molecular weight of LMP was 268.6 Kd as determined by HPGPC (Figure 1A). The composition of LMP was analyzed by HPAEC-PAD after acid hydrolysis and was found to be mainly composed of galacturonic acid (Figure 1B). The FTIR spectra of LMP consisted of characteristic peaks of polysaccharides (Figure 1C). The peak occurring in 3,432 cm^−1^ represents O-H stretching. The broad peak occurring from 1,100 to 1,070 cm^−1^ and from 1,070 to 1,043 cm^−1^ represents rhamose polygalaconic acid (Figure 1C).

### LMP Is Protective Against T1D in NOD Mice

**Figure 2:**
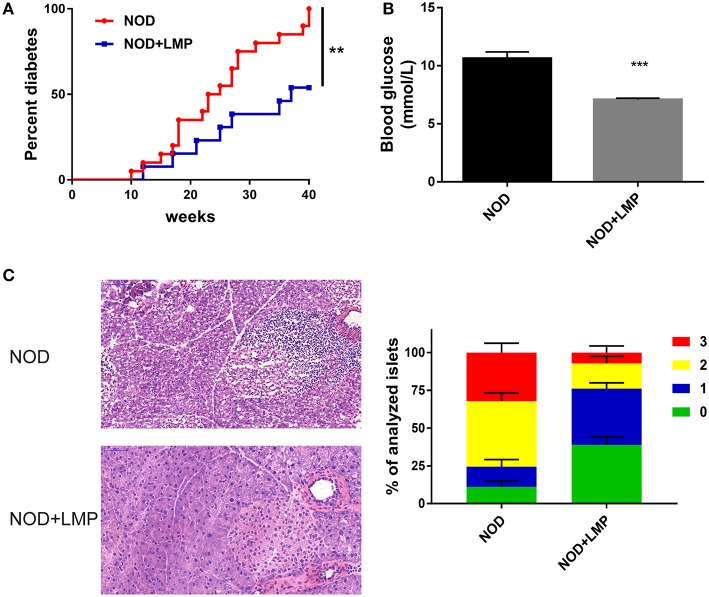
LMP supplementation protects against T1D in NOD mice. **(A)** Diabetes incidence of NOD mice treated with (LMP) or without (normal chow) 5% LMP supplementation from 4 to 40 weeks of age. *n* = 20. **(B)** The fasting blood glucose of non-diabetic NOD mice at 40 weeks of age. **(C)** H&E staining of pancreatic islets from NOD mice at 40 weeks of age. Insulitis score was quantified. Scale bar, 100 μM. ^**^p < 0.01 and ^***^p < 0.001.

To examine the protective effects of LMP supplementation on T1D development, female NOD mice were weaned onto control (AIN-93G) or LMP-supplemented diets for up to 40-weeks age. LMP supplementation prevented T1D development, as 53.8% of the LMP-fed mice (7 mice) vs. all mice (20 mice) in the control group had developed overt diabetes (Figure 2A), and fasting glucose levels of LMP-fed mice were significantly lower than control NOD mice (Figure 2B). Accordantly, less immune cell infiltration and insulitis in pancreatic islets were observed in LMP-supplemented mice as indicated by histological examination and insulitis score (Figure 2C). Taken together, our results demonstrated that LMP supplementation protects against T1D in NOD mice.

### The Protective Effect of LMP on T1D Development Is Dependent on the Caecal Microbiota

**Figure 3:**
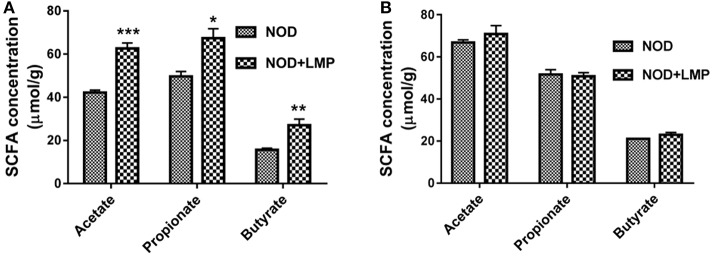
LMP increases acetate, propionate and butyrate production in the caecum of NOD mice. **(A)** Acetate, propionate, butyrate in caecal content were analyzed by GC-MS. **(B)** Acetate, propionate, butyrate in colonic content were analyzed by GC-MS. Bars represent means ± SEM; ^*^p < 0.05; ^**^p < 0.01, and ^***^p < 0.001.

Dietary prebiotic fibers would preferentially expand beneficial bacteria to produce more SCFAs and thereby impart immunomodulatory effects (28). As LMP is predominantly fermented by gut microbiota in proximal large intestine (29), we firstly determined LMP fermentation derived SCFA in the caecum and colon, and found that acetate, propionate, butyrate and total SCFAs of LMP-supplemented mice significantly increased in caecum (Figure 3A) but not in colon (Figure 3B), demonstrating that most of the LMP is fermented by caecal microbiota before it reaches the colon to exert its effect.

**Figure 4:**
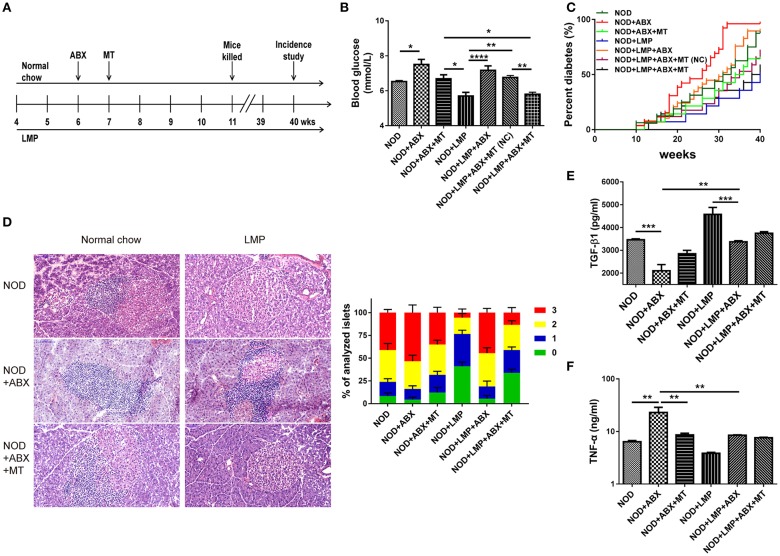
Protective effects of LMP on T1D are abolished by antibiotics (ABX)-induced abberation of gut microbiota. **(A)** Animal experiment design for ABX treatment and microbiota transfer (MT). **(B)** After ABX and MT treatment, the blood glucose was measured at 11-weeks age. **(C)** After ABX and MT treatment, the diabetes incidence was shown from 4 to 40 weeks of age. *n* = 20. **(D)** H&E staining of pancreatic islets from NOD mice at the age of 11-weeks age. Insulitis score was quantified. Scale bar, 100 μm. **(E)** The anti-inflammatory cytokine TGF-β1 was quantified in pancreas by ELISA. **(F)** The pro-inflammatory cytokine TNF-α was quantified in pancreas by ELISA. Bars represent means ± SEM; ^*^p < 0.05; ^**^p < 0.01; ^***^p < 0.001, and ^****^p < 0.0001.

In addition, dysbiosis is involved in T1D development in NOD mice (8, 16, 30). To further determine the cause or consequence of the LMP induced changes in microbiota, we performed two experiments: (i) We depleted the microbiota by ABX treatment and followed the diabetes incidence and (ii) we performed a transfer study of caecum microbiota of LMP-fed mice (MT) and normal chow-fed mice (NC) to control mice to confirm protective effects. NOD mice were treated by broad-spectrum ABX for 7 days to deplete gut microbiota, followed by 7-days MT treatment (Figure 4A). Incidence of diabetes was followed and effects of ABX treatment and MT were examined at 11-weeks age.

We found that ABX-treated mice exhibited increased blood glucose levels, and transfer of microbiota from LMP-fed donor mice (MT) afforded a better protective effect than those from normal chow-fed mice [MT(NC)] (Figure 4B). Accordantly, the incidence of T1D was lower in the NOD + LMP + ABX + MT mice compared with NOD + LMP + ABX + MT (NC) (Figure 4C). ABX treatment resulted in enhanced insulitis with increased immune cell infiltration in the pancreas (Figure 4D), upregulated proinflammatory (TNF-α) production and downregulated regulatory cytokine (TGF-β1) production (Figures 4E,F).

### LMP Prevents T1D-Associated Caecum Dysbiosis

**Figure 5:**
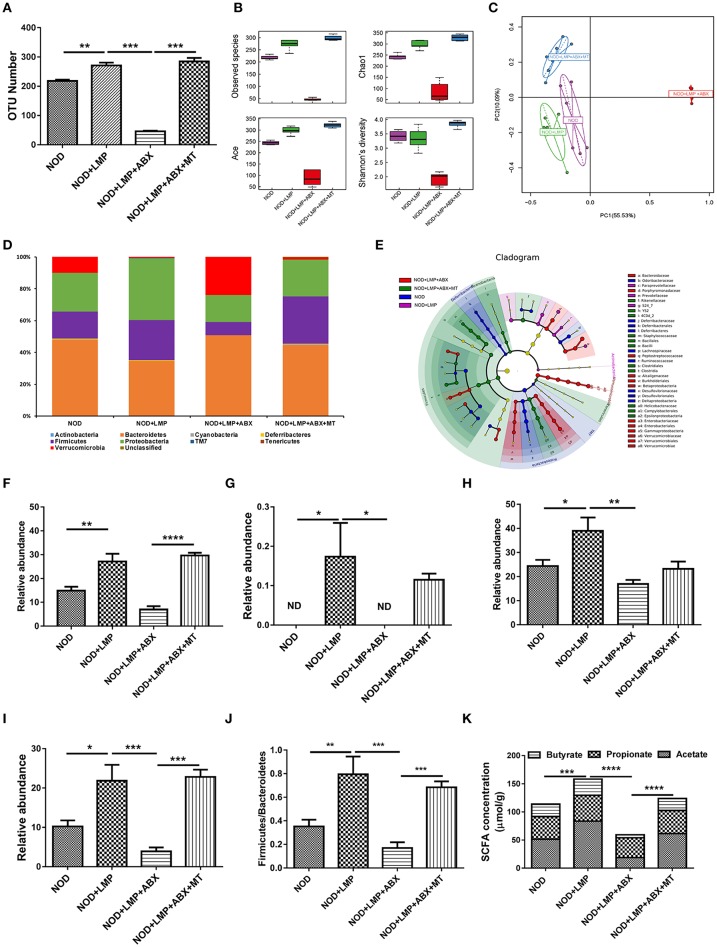
LMP prevents caecal dysbiosis of microbiota at onset of T1D. **(A)** The OTU Number among four groups of NOD mice after different treatments. **(B)** Alpha diversity indices among the four treated groups of NOD mice were displayed using boxplots. **(C)** Principal Component Analysis (PCA) analysis based on OTU abundance was shown. **(D)** The taxonomic composition distribution among four groups of phylum-level. **(E)** Cladograms generated by LEfSe indicate differences in taxa between different groups. **(F–J)** Relative abundance of *Firmicutes* **(F)**, *TM7* **(G)**, *Proteobacteria* **(H)**, *Clostridia* **(I)**, and the ratio of *Firmicutes/Bacteroidetes* **(J)** were shown. **(K)** Acetate, propionate, butyrate in caecal content were analyzed by GC-MS. Bars represent means ± SEM; ^*^p < 0.05; ^**^p < 0.01; ^***^p < 0.001, and ^****^p < 0.0001; Each point represents one mice.

Next, we investigated whether LMP modulate diversity or specific bacterial species in caecum through 16s rDNA sequencing. As shown in Figure 5, LMP supplementation increased taxonomic units (OTU) (Figure 5A), the abundance and diversity of caecal microbiota composition (Figure 5B). By principal component analysis (PCA), we found that LMP-fed mice were distinctly separated from other three groups (Figure 5C). Besides, the taxonomic composition distribution histograms of each group were analyzed at phylum level (Figure 5D), and a significant change in the phyla *Firmicutes, TM7, Proteobacteria*, and class *Clostridia* composition was observed in the different treatment by linear discriminant analysis (Figure 5E). Specifically, LMP supplementation markedly enhanced the relative abundance of microbes from the phyla *Firmicutes, TM7, Proteobacteria* and class *Clostridia*, as well as ratio of the phylum *Firmicutes* vs. *Bacteroidetes*, in NOD+LMP mice (Figures 5F–J). Consistent with the higher microbial abundance and butyrate-producing *Clostridia*, more SCFAs in caecum were observed in LMP-fed mice (Figure 5K). In all aforementioned effects, ABX treatment reversed LMP-induced effects, which could be restored with transfer of feces of LMP-fed NOD mice. Taken together, our data clearly demonstrated that LMP administration prevents the T1D associated gut dysbiosis in NOD mice.

### LMP Prevents Barrier Dysfunction and Immune Imbalance in Caecum of NOD Mice

**Figure 6:**
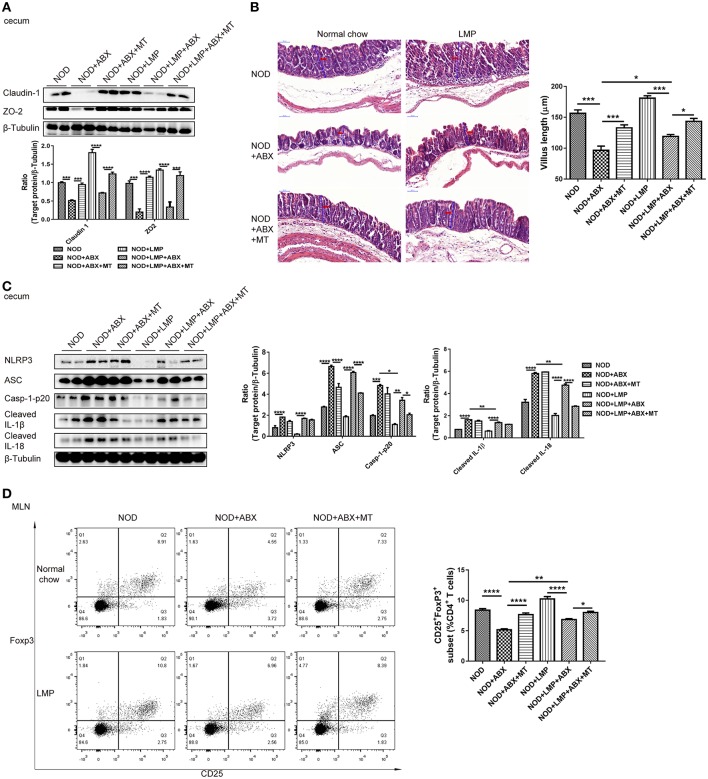
LMP-induced caecal homeostasis is microbiota-dependent. **(A)** Tight junction proteins claudin-1 and ZO-2 were detected by Western blot analysis. **(B)** H&E staining of caecum from NOD mice after different treatments at the age of 11 weeks was shown. Scale bar, 50 μm. **(C)** Inflammasome pathway proteins, NLRP3, casp-1-p20, cleaved IL-1β, and cleaved IL-18 in caecum were detected by Western blot analysis. **(D)** The frequency of CD25^+^ Foxp3^+^ cells in total of CD4^+^ T cells in MLN was analyzed by flow cytometry (*n* = 6). Bars represent means ± SEM; ^*^p < 0.05; ^**^p < 0.01; ^***^p < 0.001, and ^****^p < 0.0001.

As microbial-derived SCFAs promote gut barrier integrity and mucosal immune to reduce leakage of inflammatory mediators into systemic circulation, which protects against T1D (31). Next, we analyzed the LMP-modulated microbial balance and increased SCFAs on gut function. In the caecum, the expression of claudin-1 and ZO-2 were upregulated by LMP (Figure 6A). Accordantly, LMP supplementation significantly improved morphology of the caecum with improved barrier integrity and increased villus length in NOD mice (Figure 6B). Furthermore, downregulation of NLRP3, caspase-1-p20, cleaved-IL-1β, and IL-18 were observed in caecum of LMP-fed NOD mice compared to normal chow fed control mice (Figure 6C). Consistent with the beneficial effects on caecal function, increased frequencies of Foxp3^+^ Treg in MLN of caecum further confirmed the immunomodulatory effects of LMP (Figure 6D). However, the beneficial effects of LMP on caecum homeostasis was abolished by ABX treatment, since ABX treatment worsened caecal barrier dysfunction, morphology, and immune inflammatory response as evidenced by downregulated claudin-1 and ZO-2 expression, reduced villus length, upregulated NLRP3 inflammasome activation (NLRP3, ASC, caspase-1 p20, cleaved IL-1β, and IL-18), decreased Foxp3^+^ Treg frequencies in MLN. While transfer of caecal microbiota of LMP-fed NOD mice reversed ABX-induced caecal barrier dysfunction and immune dysregulation, indicating LMP-mediated microbial balance further stimulates gut homeostasis.

### LMP-Mediated Caecal Homeostasis Shapes Pancreatic Immune Environment in NOD Mice

**Figure 7:**
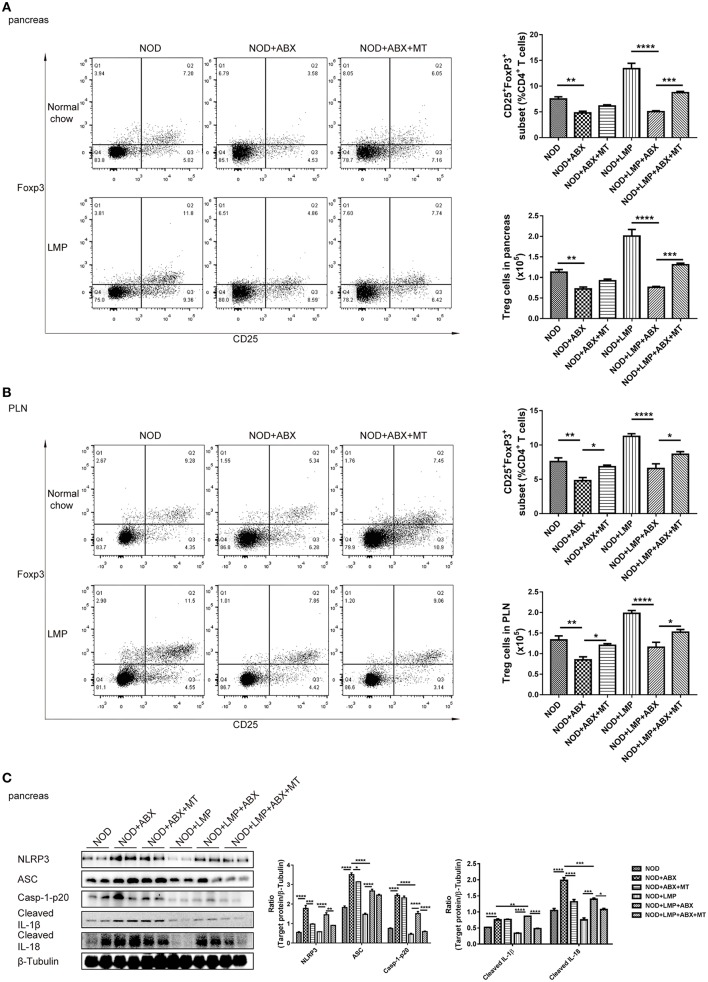
LMP-mediated caecal homeostasis shapes pancreatic immune environment in NOD mice. **(A)** The frequency of CD25^+^ Foxp3^+^ cells in total CD4^+^ T cells in pancreas was determined by flow cytometry (*n* = 6). **(B)** The frequency of CD25^+^ Foxp3^+^ cells in total CD4^+^ T cells in PLN was determined by flow cytometry (*n* = 6). **(C)** Inflammasome pathway proteins, NLRP3, casp-1-p20, cleaved IL-1β, and cleaved IL-18 in pancreas were analyzed by Western blot analysis. ^*^p < 0.05; ^**^p < 0.01; ^***^p < 0.001, and ^****^p < 0.0001.

To verify the LMP-mediated caecal homeostasis on T1D prevention, we further detected the pancreatic immune responses in NOD mice treated with LMP, ABX, and fecal microbial transplantion. Auto-immuno-attenuating effects of LMP were confirmed by the fact that frequencies of Foxp3^+^ Treg in both pancreas and PLN increased in NOD+LMP mice and decreased in ABX-treated NOD mice, but restored with the transfer of feces of LMP-fed NOD mice (Figures 7A,B). In addition, the modulatory effect of LMP on pancreatic immune responses during T1D was further confirmed by its suppressive effect on NLRP3 inflammasome activation (NLRP3, ASC, caspase-1 p20, cleaved IL-1β, and IL-18 expression) in the pancreas (Figure 7C). Meanwhile, ABX treatment promoted NLRP3 inflammasome activation in pancreas, and transfer of caecal microbiota of LMP-fed NOD mice reversed NLRP3 inflammasome activation. These results further demonstrated that LMP-mediated caecal homeostasis shapes pancreatic immune environment, thus preventing T1D development.

## Discussion

**Figure 8:**
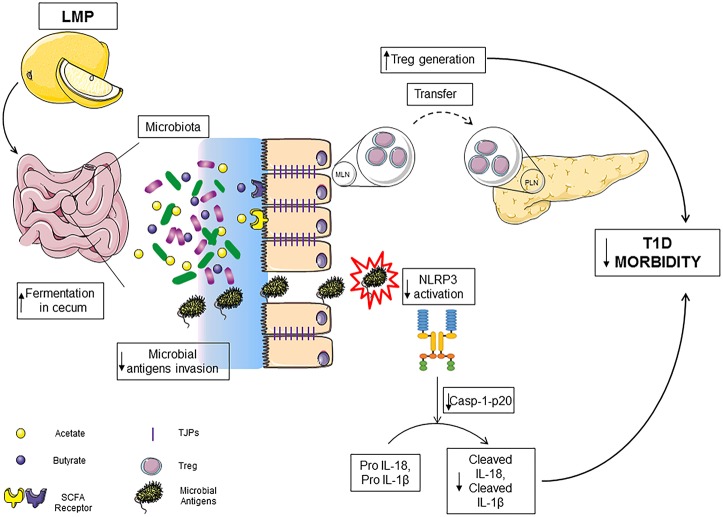
LMP supplementation limits T1D by modulating gut homeostasis in NOD mice. LMP supplementation induces gut microbiota homeostasis to increase the production of SCFA in caecum and normalizes caecal barrier function by upregulating the expression of tight junction proteins, which results in decreased microbial antigen translocation, surpressed NLRP3 inflammasome activation and enhanced frequencies of Foxp3^+^ Treg in caecum and pancreas.

Here, we demonstrate that the previously unreported LMP supplementation reduces T1D incidence in NOD mice. The effect results from a positive impact on caecal microbiota, enhanced production of immune modulating bacterial products SCFA and improved intestinal integrity in the caecum. Induced gut homeostasis by LMP results in modulated gut-pancreatic autoimmune responses and hence protection against the development of T1D (Figure 8).

In comparison to previous findings from our group (16) and others (5) on mouse models of T1D, this study demonstrated that the protective effect of dietary fibers on the development of T1D was mediated by caecal homeostasis. This location of action in the large intestine may be attributed to the low esterified chemical composition of LMP that causes its rapid fermentation in the caecum. It has been suggested that the degree of esterification in the structure of pectin affects the fermentation location, product and health-promoting effects conferred (15, 29), and LMP supplementation in rat leading to more SCFAs in caecal contents (62μmoL/g) than colonic contents (22μmoL/g) (32), while the main fermentation region of high methoxylated pectins partly shifts from the caecum to the distal colon (33).

Intestinal dysbiosis with decreased microbiota diversity and loss of beneficial intestinal micro-organisms could lead to T1D (5, 21, 34, 35). For instance, a reduced ratio of *Firmicutes* to *Bacteroidetes*, has been associated with and may contribute to the development of T1D, and microbial homeostatic maintenance has been considered as frontier strategy to treat T1D (3, 30). Diversified microbiota composition and enriched beneficial intestinal bacteria with dietary supplementation of pectins have been demonstrated earlier (29, 36). Consistent with these earlier data, LMP supplementation increased α-diversity and the ratio of *Firmicutes/Bacteroidetes* in LMP-fed NOD mice. Additionally, LMP markedly increased the relative abundance of bacterial taxa including *Firmicutes, TM7, Proteobacteria, Clostridia* in NOD mice. Enriched *Proteobacteria* and *TM7* phyla were found in mice fed gluten-free diet during pregnancy and lactation and their offspring with decreased incidence of diabetes and insulitis (37). Indigenous *Clostridium* species promoted gut and pancreatic Foxp3^+^ Treg cell accumulation (38, 39). Apparently, dietary pectin treatment significantly influences microbiota composition in NOD mice, which contributed to T1D prevention. Furthermore, experiments with ABX treatment followed by MT of caecal microbiota also confirmed that the protective effect of LMP on T1D was dependent on caecal microbiota. Notably, transferring caecal microbiota shaped by LMP into ABX-treated recipient mice fed on LMP conferred the most beneficial effects: restored caecal SCFA production, barrier function and modulating effects against T1D. When LMP-fed caecal microbiota was transferred to the ABX-treated mice fed on normal chow, only a trend of restoration was observed (Figures 4B,C), implying that LMP supplementation is essential to shape the “protective” caecal bacteria and that LMP-shaped caecal microbiota play a critical role in LMP-mediated protective effects. While ABX and MT experiments here have clearly demonstrated the role of caecal microbiota in LMP-mediated attenuation of T1D, future confirmatory experiments in germfree NOD mice may be more direct to establish its causal role.

Intriguingly, we observed higher increases in acetate, propionate and butyrate in caecum of NOD mice after LMP feeding. The production of SCFAs depend on the structure and chemical composition of dietary components and resident microbial composition (5, 29), higher increases in SCFAs production suggest that LMP may promote the growth and activity of SCFAs-producing bacteria in caecum. Indeed, *Firmucutes* phylum and *Clostridia*, which preferentially produce butyrate (5), were increased by LMP treatment. SCFAs production as a result of dietary supplementation provides a direct link to the associated beneficial effects. Specifically, enhanced butyrate production by dietary impact has been associated with modulatory effects on T1D (5, 40). Acetate, the most abundant SCFA in caecum of LMP-fed NOD mice, has also been clearly associated with modulating effects on T1D (5). Here, we provide the first evidence that diet enhanced propionate production was correlated with protection against T1D.

Foxp3^+^ Tregs, capable of suppressing proliferation and activation of autoimmune CD4^+^ and CD8^+^ effector T cells, are impaired in number and function during the development of T1D. They are the target cells in numerous studies to develop therapeutic strategies for T1D. Recently, the regulatory mechanisms of Foxp3^+^ Tregs have been unraveled in preventing over-activation of CD4^+^ or CD8^+^ T effector cells, that is through a variety of activities including cell-cell contact and soluble factors (41, 42). SCFAs elevate the number and function of induced Foxp3^+^ Treg cells in the colon and peripheral blood (43, 44). Furthermore, SCFAs also influence gene transcription in Foxp3^+^ Treg cells (45) via inhibition of histone-deacetylase (HDAC) activity. Consistent with these earlier data, significant increase of CD4^+^CD25^+^Foxp3^+^ T cells in pancreas, PLN and caecal MLN was observed in LMP-treated NOD mice, which explained its effects on delaying the onset of T1D. Whereas, our data clearly indicate caecal immune balance induced by LMP positively influenced pancreatic autoimmunity, the exact mechanism underlying caecum-pancreas axis that how Foxp3^+^ Tregs are induced from caecum and migrate or influence pancreatic immune environment is still unclear and deserve further investigations.

Recently, the role of NLRP3 inflammasome in T1D has been increasingly recognized (46, 47). Genetic ablation of NLRP3 protects mice from the development of T1D by suppressing T-cell activation and Th1 differentiation as well as by modulating pathogenic T-cell migration to the pancreatic islet. These effects of NLRP3 deficiency were mediated by modulating chemokine receptors on T cells and chemokine expression in the islets (47). In the settings of intestinal microenvironment, however, a dual role of NLRP3 inflammasome has been unraveled. While some studies showed a regulatory role of NLRP3 in maintaining immune tolerance and epithelial barrier integrity (48), others reported that overactivation of NLRP3 inflammasome under inflamed condition is associated with immune imbalance and detrimental effects (49). In this context, canonical vs. non-canonical NLRP3 inflammasome activation pathways associated with different downstream effector molecules are currently being investigated (49). Also different roles of NLRP3 in regulating intestinal homeostasis may be ascribed to different experimental models and settings. LPS induced activation of NLRP3 inflammasome was accompanied with intestinal epithelial dysfunction, which can be significantly inhibited by SCFAs (50). Dysfunction of intestinal epithelial barrier is suggested to be a crucial factor in the onset of intestinal and systemic autoimmune diseases, including T1D (51). In our observation, during the development of T1D with loss of intestinal homeostasis, LMP supplementation suppressed NLRP3 activation, which was associated with attenuated intestinal permeability, enhanced tight-junction protein expression and thus barrier function. Whereas, our data supported a positive association of canonical NLRP3 activation with the development of T1D and dysregulated intestinal homeostasis, molecular mechanisms to demonstrate any causative link remained to be explored.

In summary, we demonstrate that a novel dietary fiber, low esterified pectin, can ameliorate T1D through selective enrichment of specific microbiota species that produce SCFAs. This has specific effects on inflammasome activation, gut integrity and immune cell populations in the gut and pancreas, which prevent the development of T1D.

## Data Availability

The datasets generated for this study can be found in NCBI PRJNA532521, PRJNA532521.

## Ethics Statement

All animal experiments were carried out according to protocols approved by the Institutional Animal Ethics Committee of Jiangnan University (JN. No: 20150331-0410), in compliance with the recommendations of national and international guidelines for the Care and Use of Laboratory Animals, and were performed in accordance with the guidelines therein.

## Author Contributions

CW, WN, XF, WL, JL, and HL performed experiments and analyzed data. L-LP, WC, HZ, JL, BA, and PdV provided intellectual inputs, contributed to the data acquisition and critically reviewed the manuscript. JS, L-LP, and PdV designed and interpreted experiments. XP, JS, PdV, and L-LP wrote the paper.

### Conflict of Interest Statement

The authors declare that the research was conducted in the absence of any commercial or financial relationships that could be construed as a potential conflict of interest.

## Abbreviations

T1D: Type 1 diabetes
LMP: Low-methoxyl pectin
Treg: Regulatory T cell
MT: Microbiota transfer
NLRP3: NOD like receptor protein 3
PLN: Pancreatic lymph node
MLN: Mesenteric lymph nodes
HPGPC: High performance gel permeation chromatography
HPAEC-PAD: High performance anion exchange chromatography with pulsed amperometric detection
FTIR: Fourier transform infrared spectroscopy.

## References

[1] Atkinson MA Eisenbarth GS Michels AW. Type 1 diabetes. Lancet. (2014) 383:69–82. 10.1016/S0140-6736(13)60591-7 23890997 PMC4380133

[2] Mejia-Leon ME Barca AM. Diet, microbiota and immune system in type 1 diabetes development and evolution. Nutrients. (2015) 7:9171–84. 10.3390/nu7115461 26561831 PMC4663589

[3] Wen L Ley RE Volchkov PY Stranges PB Avanesyan L Stonebraker AC. Innate immunity and intestinal microbiota in the development of Type 1 diabetes. Nature. (2008) 455:1109–13. 10.1038/nature07336 18806780 PMC2574766

[4] Li X Atkinson MA The role for gut permeability in the pathogenesis of type 1 diabetes–a solid or leaky concept? Pediatr Diabetes. (2015) 16:485–92. 10.1111/pedi.12305 26269193 PMC4638168

[5] Mariño E Richards JL McLeod KH Stanley D Yap YA Knight J Gut microbial metabolites limit the frequency of autoimmune T cells and protect against type 1 diabetes. Nat Immunol. (2017) 18:552 10.1038/ni.3713 28346408

[6] Paun A Yau C Danska JS. Immune recognition and response to the intestinal microbiome in type 1 diabetes. J Autoimmun. (2016) 71:10–8. 10.1016/j.jaut.2016.02.004 26908163

[7] Hu Y Wong FS Wen L. Antibiotics, gut microbiota, environment in early life and type 1 diabetes. Pharmacol Res. (2017) 119:219–26. 10.1016/j.phrs.2017.01.034 28188825 PMC5392439

[8] Knip M Siljander H. The role of the intestinal microbiota in type 1 diabetes mellitus. Nat Rev Endocrinol. (2016) 12:154–67. 10.1038/nrendo.2015.218 26729037

[9] Knip M Honkanen J. Modulation of type 1 diabetes risk by the intestinal microbiome. Curr Diab Rep. (2017) 17:105. 10.1007/s11892-017-0933-9 28942491

[10] Vaarala O Atkinson MA Neu J. The “perfect storm” for type 1 diabetes: the complex interplay between intestinal microbiota, gut permeability, and mucosal immunity. Diabetes. (2008) 57:2555–62. 10.2337/db08-0331 18820210 PMC2551660

[11] Vaarala O. Is the origin of type 1 diabetes in the gut? Immunol Cell Biol. (2012) 90:271–6. 10.1038/icb.2011.115 22290506

[12] Belizario JE Napolitano M. Human microbiomes and their roles in dysbiosis, common diseases, and novel therapeutic approaches. Front Microbiol. (2015) 6:1050. 10.3389/fmicb.2015.01050 26500616 PMC4594012

[13] Koh A De Vadder F Kovatcheva-Datchary P Backhed F. From dietary fiber to host physiology: short-chain fatty acids as key bacterial metabolites. Cell. (2016) 165:1332–45. 10.1016/j.cell.2016.05.041 27259147

[14] Sun Y He Y Wang F Zhang H de Vos P Sun J. Low-methoxyl lemon pectin attenuates inflammatory responses and improves intestinal barrier integrity in caerulein-induced experimental acute pancreatitis. Mol Nutr Food Res. (2017) 61:1600885. 10.1002/mnfr.201600885 27921358

[15] Sahasrabudhe NM Beukema M Tian L Troost B Scholte J Bruininx E. Dietary fiber pectin directly blocks toll-like receptor 2-1 and prevents doxorubicin-induced ileitis. Front Immunol. (2018) 9:383. 10.3389/fimmu.2018.00383 29545800 PMC5839092

[16] Chen K Chen H Faas MM de Haan BJ Li J Xiao P. Specific inulin-type fructan fibers protect against autoimmune diabetes by modulating gut immunity, barrier function and microbiota homeostasis. Mol Nutr Food Res. (2017) 61:1601006. 10.1002/mnfr.201601006 28218451

[17] He Y Wu C Li J Li H Sun Z Zhang H. Inulin-type fructans modulates pancreatic-gut innate immune responses and gut barrier integrity during experimental acute pancreatitis in a chain length-dependent manner. Front Immunol. (2017) 8:1209. 10.3389/fimmu.2017.01209 29018453 PMC5622924

[18] Jeurink PV van Esch BC Rijnierse A Garssen J Knippels LM. Mechanisms underlying immune effects of dietary oligosaccharides. Am J Clin Nutr. (2013) 98:572S−7S. 10.3945/ajcn.112.038596 23824724

[19] Gruber C van Stuijvenberg M Mosca F Moro G Chirico G Braegger CP. Reduced occurrence of early atopic dermatitis because of immunoactive prebiotics among low-atopy-risk infants. J Allergy Clin Immunol. (2010) 126:791–7. 10.1016/j.jaci.2010.07.022 20832848

[20] Vaarala O. The gut as a regulator of early inflammation in type 1 diabetes. Curr Opin Endocrinol Diabetes Obes. (2011) 18:241–7. 10.1097/MED.0b013e3283488218 21681088

[21] Llewellyn SR Britton GJ Contijoch EJ Vennaro OH Mortha A Colombel JF. Interactions between diet and the intestinal microbiota alter intestinal permeability and colitis severity in mice. Gastroenterology. (2017) 154:S0016508517363886. 10.1053/j.gastro.2017.11.030 29174952 PMC5847454

[22] Shohei Y Kosuke Y Shinji K Daisuke K Mako Y-Y Naoko T Role of dietary amino acid balance in diet restriction-mediated lifespan extension, renoprotection, and muscle weakness in aged mice. Aging Cell. (2018) 17:e12796 10.1111/acel.12796 29943496 PMC6052467

[23] Shingo H Kei S Hiroyuki B Hirokazu K Seiichiro A Tatsuya M Low-methoxyl pectin stimulates small intestinal mucin secretion irrespective of goblet cell proliferation and is characterized by jejunum Muc2 upregulation in rats. J Nutr. (2013) 143:34–40. 10.3945/jn.112.167064 23173170

[24] Shtriker MG Hahn M Taieb E Nyska A Moallem U Tirosh O. Fenugreek galactomannan and citrus pectin improve several parameters associated with glucose metabolism, and modulate gut microbiota in mice. Nutrition. (2017) 46:134–42.e3. 10.1016/j.nut.2017.07.012 28993009

[25] Adam CL Williams PA Garden KE Thomson LM Ross AW. Dose-dependent effects of a soluble dietary fibre (pectin) on food intake, adiposity, gut hypertrophy and gut satiety hormone secretion in rats. PLoS ONE. (2015) 10:e0115438. 10.1371/journal.pone.0115438 25602757 PMC4300082

[26] Sun J Furio L Mecheri R van der Does AM Lundeberg E Saveanu L. Pancreatic beta-cells limit autoimmune diabetes via an immunoregulatory antimicrobial peptide expressed under the influence of the gut microbiota. Immunity. (2015) 43:304–17. 10.1016/j.immuni.2015.07.013 26253786

[27] Hansen CH Krych L Nielsen DS Vogensen FK Hansen LH Sorensen SJ. Early life treatment with vancomycin propagates Akkermansia muciniphila and reduces diabetes incidence in the NOD mouse. Diabetologia. (2012) 55:2285–94. 10.1007/s00125-012-2564-7 22572803

[28] Dwivedi M Kumar P Laddha NC Kemp EH. Induction of regulatory T cells: a role for probiotics and prebiotics to suppress autoimmunity. Autoimmun Rev. (2016) 15:379–92. 10.1016/j.autrev.2016.01.002 26774011

[29] Tian L. Effects of pectin supplementation on the fermentation patterns of different structural carbohydrates in rats. Mol Nutr Food Res. (2016) 60:2256–66. 10.1002/mnfr.201600149 27174558

[30] Alkanani AK Hara N Gottlieb PA Ir D Zipris D. Alterations in intestinal microbiota correlate with susceptibility to type 1 diabetes. Diabetes. (2015) 64:db141847. 10.2337/db14-1847 26068542 PMC4587635

[31] Li N Hatch M Wasserfall CH Douglas-Escobar M Atkinson MA Schatz DA. Butyrate and type 1 diabetes mellitus: can we fix the intestinal leak? J Pediatr Gastroenterol Nutr. (2010) 51:414–7. 10.1097/MPG.0b013e3181dd913a 20706153

[32] Zhang J Lupton JR. Dietary fibers stimulate colonic cell proliferation by different mechanisms at different sites. Nutr Cancer. (1994) 22:267–76. 10.1080/01635589409514352 7877896

[33] Gerhard D Angelika L Jürgen P The degree of methylation influences the degradation of pectin in the intestinal tract of rats and *in vitro*. J Nutr. (2002) 132:1935–44. 10.1093/jn/132.7.1935 12097673

[34] Paun A Yau C Danska JS. The influence of the microbiome on type 1 diabetes. J Immunol. (2017) 198:590–5. 10.4049/jimmunol.1601519 28069754

[35] Chung WSF Walker AW Louis P Parkhill J Vermeiren J Bosscher D. Modulation of the human gut microbiota by dietary fibres occurs at the species level. BMC Biol. (2016) 14:3. 10.1186/s12915-015-0224-3 26754945 PMC4709873

[36] Tian L Bruggeman G van den Berg M Borewicz K Scheurink AJ Bruininx E. Effects of pectin on fermentation characteristics, carbohydrate utilization and microbial community composition in the gastrointestinal tract of weaning pigs. Mol Nutr Food Res. (2016) 61:1600186. 10.1002/mnfr.201600186 27198846

[37] Hansen CH Krych L Buschard K Metzdorff SB Nellemann C Hansen LH. A maternal gluten-free diet reduces inflammation and diabetes incidence in the offspring of NOD mice. Diabetes. (2014) 63:2821–32. 10.2337/db13-1612 24696449

[38] Atarashi K Tanoue T Shima T Imaoka A Kuwahara T Momose Y. Induction of colonic regulatory T cells by indigenous Clostridium species. Science. (2011) 331:337–41. 10.1126/science.1198469 21205640 PMC3969237

[39] Jia L Shan K Pan LL Feng N Lv Z Sun Y. Clostridium butyricum CGMCC0313.1 protects against autoimmune diabetes by modulating intestinal immune homeostasis and inducing pancreatic regulatory T cells. Front Immunol. (2017) 8:1345. 10.3389/fimmu.2017.01345 29097999 PMC5654235

[40] Endesfelder D Engel M Davis-Richardson AG Ardissone AN Achenbach P Hummel S. Towards a functional hypothesis relating anti-islet cell autoimmunity to the dietary impact on microbial communities and butyrate production. Microbiome. (2016) 4:17. 10.1186/s40168-016-0163-4 27114075 PMC4845316

[41] Bluestone JA Buckner JH Fitch M Gitelman SE Gupta S Hellerstein MK. Type 1 diabetes immunotherapy using polyclonal regulatory T cells. Sci Transl Med. (2015) 7:315ra189. 10.1126/scitranslmed.aad4134 26606968 PMC4729454

[42] Tree TIM Lawson J Edwards H Skowera A Arif S Roep BO. Naturally arising human CD4 T-cells that recognize islet autoantigens and secrete interleukin-10 regulate proinflammatory T-cell responses via linked suppression. Diabetes. (2010) 59:1451–60. 10.2337/db09-0503 20299476 PMC2874706

[43] Furusawa Y Obata Y Fukuda S Endo TA Nakato G Takahashi D. Commensal microbe-derived butyrate induces the differentiation of colonic regulatory T cells. Nature. (2013) 504:446–50. 10.1038/nature12721 24226770

[44] Arpaia N Campbell C Fan X Dikiy S van der Veeken J deRoos P. Metabolites produced by commensal bacteria promote peripheral regulatory T-cell generation. Nature. (2013) 504:451–5. 10.1038/nature12726 24226773 PMC3869884

[45] Zhang Y Maksimovic J Naselli G Qian J Chopin M Blewitt ME. Genome-wide DNA methylation analysis identifies hypomethylated genes regulated by FOXP3 in human regulatory T cells. Blood. (2013) 122:2823–36. 10.1182/blood-2013-02-481788 23974203 PMC3798997

[46] Pontillo A Brandao L Guimaraes R Segat L Araujo J Crovella S. Two SNPs in NLRP3 gene are involved in the predisposition to type-1 diabetes and celiac disease in a pediatric population from northeast Brazil. Autoimmunity. (2010) 43: 583–9. 10.3109/08916930903540432 20370570

[47] Hu C Ding H Li Y Pearson JA Zhang X Flavell RA. NLRP3 deficiency protects from type 1 diabetes through the regulation of chemotaxis into the pancreatic islets. Proc Natl Acad Sci USA. (2015) 112:11318–23. 10.1073/pnas.1513509112 26305961 PMC4568693

[48] Hirota SA Ng J Lueng A Khajah M Parhar K Li Y. NLRP3 inflammasome plays a key role in the regulation of intestinal homeostasis. Inflamm Bowel Dis. (2011) 17:1359–72. 10.1002/ibd.21478 20872834 PMC3026862

[49] Pellegrini C Antonioli L Lopezcastejon G Blandizzi C Fornai M. Canonical and non-canonical activation of NLRP3 inflammasome at the crossroad between immune tolerance and intestinal inflammation. Front Immunol. (2017) 8:36. 10.3389/fimmu.2017.00036 28179906 PMC5263152

[50] Feng Y Wang Y Wang P Huang Y Wang F. Short-chain fatty acids manifest stimulative and protective effects on intestinal barrier function through the inhibition of NLRP3 inflammasome and autophagy. Cell Physiol Biochem. (2018) 49:190–205. 10.1159/000492853 30138914

[51] Suzuki T. Regulation of intestinal epithelial permeability by tight junctions. Cell Mol Life Sci. (2013) 70:631–59. 10.1007/s00018-012-1070-x 22782113 PMC11113843

