# Supplementary material for: Modulation of Gut Microbiota by Low Methoxyl Pectin Attenuates Type 1 Diabetes in Non-obese Diabetic Mice
Source: Front Immunol. 2019 Jul 30;10:1733. doi: 10.3389/fimmu.2019.01733 (PMC6682655; doi:10.3389/fimmu.2019.01733)
Supplement: Supplementary file 1 [file Table_1.pdf]

Supplementary Table 1. Antibodies and reagents used for flow cytometry

Anti-	Dilution	Manufacturer	Cat. #:
CD45	1:20	BD Pharmingen	553079
CD4	1:20	BD Pharmingen	552775
CD25	1:20	BD Pharmingen	557192
Foxp3	1:50	ebioscience	12-5773
Transcription Factor Buffer Set		BD Pharmingen	562574
